# Supplementary material for: Self-Reflection and Peer-Assessments Effect on Pharmacy Students’ Performance at Simulated Counselling Sessions
Source: Pharmacy (Basel). 2022 Dec 27;11(1):5. doi: 10.3390/pharmacy11010005 (PMC9844287; doi:10.3390/pharmacy11010005)
Supplement: Supplementary file 1 [file pharmacy-11-00005-s001.zip › pharmacy-2092869-supplementary.pdf]

Student Focus Group Guide

Aim: To evaluate the PHAR3825 unit of study

SCRIPT: “Thank you for volunteering to take part in a focus group on your experience in PHAR3825 in semester 2. We anticipate the discussion to take approximately 30 minutes. I would like to remind you that the focus group is being audio recorded. All participants will be referred to by code in order to maintain anonymity.”

[Hand out numbers to students]

QUESTIONS TO ASK

a. I am going to focus on the counselling process of PHAR3825, not the dispensing labels. This includes the simulated cases for counselling, self-reflection of your counselling sessions and the peer feedback of received for your counselling. How do you feel about these different aspects?
    i. What do you think about the simulated cases?
    ii. What do you think about the self-reflection process?
    iii. What do you think about the peer feedback process?

b. What do you think about using these techniques as a learning tool?
    i. Compared to other ways you have been taught in past units?
    ii. Do you think it was more or less effective?

c. The therapeutic units have a larger focus on group learning versus individual learning, In your opinion, how do you think this programme compares to other units you have participated in?
    i. What are the differences for you as a pharmacy student?
    ii. Do you have a preference? Why?

d. Has PHAR3825 had an impact on your placements or workplace?

e. A goal of the counselling process has been to assist you in practicing and displaying empathy. What has your experience been with this in the classroom setting and in placements or the workplace?

f. What concerns do you have about the counselling process in PHAR3825 and how could they be improved?
